# Epidural Dexamethasone for Postoperative Analgesia in Patients Undergoing Unilateral Inguinal Herniorrhaphy: A Comparative Study

**DOI:** 10.1155/2017/7649458

**Published:** 2017-02-28

**Authors:** M. R. Razavizadeh, MR. Fazel, N. Heydarian, F. Atoof

**Affiliations:** ^1^Trauma Research Center, Kashan University of Medical Sciences, Kashan, Iran; ^2^Department of Anesthesiology, Kashan University of Medical Sciences, Kashan, Iran; ^3^Department of Community Medicine, Kashan University of Medical Sciences, Kashan, Iran

## Abstract

*Background*. This study was designed to evaluate the effect of adding dexamethasone to epidural bupivacaine on postoperative analgesia in unilateral inguinal herniorrhaphy.* Methods*. Forty-four patients were enrolled in this double-blind, clinical trial study. Patients were randomly allocated into dexamethasone or control group. In the dexamethasone group, patients received 18 ml of bupivacaine 0.5% and 2 ml (8 mg) of dexamethasone; in the control group, patients received 18 ml of bupivacaine 0.5% and 2 ml of normal saline. The onset of sensory block and its duration and incidence of nausea and vomiting were recorded.* Results*. The onset of epidural anesthesia was significantly more rapid in the dexamethasone group than in the control group (*P* < 0.001). Duration of analgesia was markedly prolonged in the dexamethasone group than in the control group (*P* < 0.001). Five patients (22.7%) in the control group had nausea in the first hour after the procedure (*P* = 0.048). None of the patients in the dexamethasone group had nausea. None of our patients had vomiting in the two groups.* Conclusions*. This study showed that adding dexamethasone to bupivacaine significantly prolongs the duration of postoperative analgesia. This trial is registered with Iranian Registry of Clinical Trials (IRCT) number IRCT2012062910137N1.

## 1. Introduction

Uncontrolled postoperative pain may produce a range of harmful effects [1]. Attenuation of postoperative pain may decrease perioperative mortality and morbidity [2]. Epidural anesthesia and analgesia are a safe and effective method for control of postoperative pain [3]. Prolonging the duration of local anesthesia is often desirable because it produces analgesia in the postoperative period.

Various adjuvants have been used to prolong epidural anesthesia. The combination of epidural opioid and local anesthetic provides good pain control during the first postoperative day but is associated with nausea, vomiting, sedation, pruritus, urinary retention, and respiratory depression [4].

Several studies reveal that the addition of dexamethasone to bupivacaine significantly prolongs the duration of the motor block and improves the quality of analgesia following interscalene and supraclavicular block [5–9]. Another study revealed that epidural bupivacaine-dexamethasone mixture had almost the same analgesic potency as bupivacaine-fentanyl with opioid-sparing and antiemetic effects [10].

In this study, we evaluated the effect of adding dexamethasone to epidural bupivacaine on the onset anesthesia and duration of analgesia after unilateral inguinal herniorrhaphy.

## 2. Methods

Forty-four patients undergoing elective unilateral inguinal herniorrhaphy, class 1 or 2 of the ASA (American Society of Anesthesiology), were admitted to Shahid Beheshti Hospital of Kashan University of Medical Sciences (KAUMS) from May 2012 to March 2013 and were enrolled in this randomized, double-blind, clinical trial study. After institutional approval and approval by ethics committee of the university, informed consent was obtained from each patient before inclusion in the study.

Patients with complicated inguinal hernia (e.g., incarcerated and strangulated), peptic ulcer disease, diabetes mellitus, coagulopathies, skin infection on lumbar spine, severe or morbid obesity, and renal or liver disease, those with allergy to local anesthetics, and those on long-term steroid therapy were excluded from the study.

Patients were randomly allocated into dexamethasone or control group using block randomization. Patients and an anesthesiologist who performed epidural anesthesia were blind to the patients' group allocation. After arrival at the operating room, the study protocol and the epidural procedure were explained to each patient. Standard monitoring (pulse oximetry, electrocardiography, and noninvasive blood pressure monitoring) was established. Intravenous access was secured and each patient received 10 ml/kg of intravenous normal saline during 30 minutes.

Single-shot epidural anesthesia was performed under aseptic conditions with patients in the sitting position, using “loss of resistance technique” and “18 G Tuohy needle,” at the L2-3 level, after skin infiltration with lidocaine 2%. A test dose of 3 ml lidocaine 2% and epinephrine 1 : 200000 was injected to rule out intravenous or subarachnoid injection.

In the dexamethasone group, patients received 18 ml of isobaric bupivacaine 0.5% and 2 ml (8 mg) of dexamethasone, and in the control group, patients received 18 ml of isobaric bupivacaine 0.5% and 2 ml of normal saline epidurally. The end time of injection, the onset of sensory block with the pinprick test at the site of surgery, presence or absence of nausea, and vomiting in the first hour after the procedure were noted. After the operation, the onset of pain perception by the patients at the site of surgery was also recorded.

The onset of epidural anesthesia was defined as the time interval between the end of drug injection and the total abolition of pinprick response at the site of surgery. The duration of epidural analgesia was defined as the time from onset of sensory block up to pain perception at the site of surgery by the patient at rest with the moving. Any unwanted complication was recorded.

### 2.1. Sample Size

With regard to the onset of anesthesia in both groups, with and without dexamethasone (14.5 ± 2.10 versus 18.15 ± 4.25 min, resp.), based on previous studies [6], and with a power of 80% and a significance level of 95%, a sample size of 22 patients in each group was determined.

### 2.2. Data Analysis

Statistical analyses were performed using Statistical Package for Social Science (SPSS) for Windows version 19.0. Statistical tests such as Student's *t*-test, the Mann–Whitney *U*-test, and the chi-square test were used to assess significant differences between the two groups.

## 3. Results

Forty-four patients were studied in the two groups, 22 in each group. Demographic data and duration of surgery were similar in both groups and all patients were males (Table 1).

The onset of anesthesia was significantly more rapid in the dexamethasone group than in the control group (*P* < 0.001). The duration of analgesia was markedly prolonged in the dexamethasone group than in the control group (*P* < 0.001) (Table 2).

Only five (22.7%) of the patients had nausea in the first hour after the procedure, and all were in the control group (*P* = 0.048). None of the patients in the dexamethasone group had nausea. None of the patients in either group had vomiting (Table 2).

## 4. Discussion

In recent studies, steroids were added to local anesthetics to enhance the duration of anesthesia and improve the quality of analgesia in regional blocks. This combination was used for subcutaneous infiltration, neuraxial route, and peripheral nerve blocks [7, 11, 12].

In the study of Biradar et al. [13] in 2013, the addition of dexamethasone (8 mg) to the combination of lidocaine 1.5% and epinephrine 1 : 200000 in a supraclavicular block substantially prolonged the duration of sensory and motor block (326.0 ± 58.6 versus 159.0 ± 20.1 and 290.6 ± 52.7 versus 135.5 ± 20.3 min, resp.).

Kopacz et al. [14] showed that the combination of bupivacaine and dexamethasone produces a prolonged duration of anesthesia and analgesia in intercostal blockade. In a randomized, controlled clinical study, Bigat et al. [15] added 8 mg dexamethasone to lidocaine for intravenous regional anesthesia (IVRA) in patients undergoing hand surgery. They concluded that the addition of dexamethasone to lidocaine for IVRA improves postoperative analgesia during the first postoperative day.

In another study, Holte et al. [16] demonstrated that subcutaneous injection of bupivacaine with dexamethasone prolonged local analgesia compared with bupivacaine alone. Furthermore, it has been shown that the addition of other steroids such as methylprednisolone to local anesthetics in an axillary block considerably prolongs the duration of analgesia and motor block [17].

The combination of dexamethasone and local anesthetic has been used in the epidural space in some previous studies in different manners. In order to assess the net effect of adding dexamethasone to epidural local anesthetic on the duration of postoperative analgesia, we only added dexamethasone to epidural bupivacaine, and all our patients were male and candidates for unilateral inguinal herniorrhaphy. We showed that adding dexamethasone to epidural bupivacaine significantly prolongs the duration of perioperative analgesia, in addition to producing earlier onset of action.

Shrestha et al. [7] in 2007 compared addition of dexamethasone (8 mg) or tramadol (2 mg/kg) to epidural bupivacaine, and the duration of postoperative analgesia was substantially more in the dexamethasone group than in the tramadol group (1028 and 453 min, resp.).

The study of Naghipour et al. [18] in 2013 has indicated that the addition of dexamethasone (8 mg and 4 mg in lumbar and thoracic epidural catheterization, resp.) to bupivacaine and fentanyl for postoperative epidural analgesia results in an increased duration of analgesia (372 ± 58.1 versus 234.6 ± 24.3 min). The pain score and pentazocine use in the dexamethasone group were less than those in the control group (37.1 ± 19.7 mg versus 73.1 ± 17.6 mg, resp.; *P* = 0.001). Although the study used bupivacaine and dexamethasone only for postoperative analgesia, the results confirm the results of our study.

Hefni et al. [19] evaluated the efficiency and safety of different doses of epidural dexamethasone for postoperative analgesia. Patients received 10 ml epidural plain bupivacaine 0.25% in the control group with 4 mg, 6 mg, and 8 mg dexamethasone in the other groups. After surgery, the time to first analgesic requirement was significantly prolonged in the dexamethasone groups compared with the control group. There was a significant reduction in postoperative meperidine consumption during the first 24 h in the dexamethasone groups in comparison with the control group. The visual analogue scale (VAS) scores were significantly lower and the patient satisfaction score was significantly higher in the dexamethasone groups compared with the control group. Similar to our study, this finding showed that epidural dexamethasone reduces postoperative pain following surgery.

Results of our study showed that the complete sensory block in the dexamethasone group was earlier than in the control group (7.64 ± 2.74 versus 12.09 ± 2.79 min).

Yadav et al. [9] in 2008 used 24 ml lidocaine with 4 mg dexamethasone (*n* = 30) or neostigmine (*n* = 30) compared with a control group (*n* = 30) in brachial plexus block in 90 patients; the onset was better in the dexamethasone group. The earlier onset of action in the steroid group may be due to the synergistic action of dexamethasone with local anesthetics on the blockage of nerve fibers [20, 21].

The mechanisms by which dexamethasone increased the duration of nerve blockade and analgesic effect are not fully understood, though it is commonly attributed to anti-inflammatory and immunosuppressive actions. This is supported by the finding that the block length is increased by glucocorticoid potency and is completely reversed by administration of a specific glucocorticoid receptor antagonist [21, 22].

According to the traditional theory of steroid action, glucocorticoids bind to intracellular receptors and modify nuclear transcription [23]. In our study, dexamethasone had a relatively rapid effect that cannot be explained by the above mechanism.

Corticosteroids may have a local effect on the nerve. It has been shown that local steroid application suppresses transmission in thin unmyelinated nociceptive C-fibers, but not in myelinated A-beta fibers [24]. The dexamethasone effect in our study may be related to this action.

Furthermore, epidural dexamethasone may suppress prostaglandin formation in the spinal cord. Peripheral tissue damage during surgery activates phospholipase A2 and upregulates the expression of cyclooxygenase-2 in the spinal cord, leading to prostaglandin synthesis and a resultant hyperalgesic state [25]. Inflammatory, metabolic, hormonal, and immune responses to surgery are activated immediately after the surgical incision, so preoperative administration of steroids may reduce these responses, by their anti-inflammatory and immunosuppressive effects and by inhibiting both phospholipase A2 and cyclooxygenase-2 enzymes [26].

Five (22.7%) of our patients in the control group had nausea in the first hour after the procedure. None of the patients in the dexamethasone group had nausea. This result may be due to the antiemetic effect of dexamethasone, which has been demonstrated in other studies using perioperative corticosteroids [27–29].

Adverse effects with a single dose of dexamethasone are extremely rare and slight in nature, and prior studies have demonstrated that short-term (<24 hour) use of dexamethasone is safe [30, 31].

We used a dose of 8 mg dexamethasone in lumbar epidural anesthesia in our patients because administration of this dose was deemed to be safe in adults; however, additional studies must be done to determine the optimal dose of epidural dexamethasone.

## Figures and Tables

**Table 1:** Demographic parameters in two groups.

Characteristics	Dexamethasone group (*n* = 22)	Control group (*n* = 22)	*P* value
Age (year)	46.09 ± 13.34	48.64 ± 13.83	0.54
ASA I (number)	21 (95.5%)	20 (90.9%)	0.55
ASA II (number)	1 (4.5%)	2 (9.1%)	0.55
Duration of surgery (minute)	76.3 ± 12.7	78.1 ± 12.4	0.55

**Table 2:** Clinical variables in two groups.

Characteristics	Dexamethasone group (*n* = 22)	Control group (*n* = 22)	*P* value
Onset of epidural anesthesia (minute)	7.64 ± 2.74	12.09 ± 2.79	<0.001
Duration of analgesia (minute)	692.55 ± 245.88	286.59 ± 84.02	<0.001
Nausea	0	5 (22.7%)	0.048
Vomiting	0	0	—
